# Probabilistic Flexural Fatigue in Plain and Fiber-Reinforced Concrete

**DOI:** 10.3390/ma10070767

**Published:** 2017-07-07

**Authors:** José D. Ríos, Héctor Cifuentes, Rena C. Yu, Gonzalo Ruiz

**Affiliations:** 1 Grupo de Estructuras, ETS de Ingeniería, Universidad de Sevilla, Camino de los Descubrimientos, s/n, 41092 Sevilla, Spain; jdrios@us.es; 2 ETSI de Caminos, Canales y Puertos, Universidad de Castilla-La Mancha, Avenida Camilo José Cela, s/n, 13071 Ciudad Real, Spain; rena@uclm.es (R.C.Y.); Gonzalo.Ruiz@uclm.es (G.R.)

**Keywords:** probabilistic fatigue model, flexural fatigue, fiber-reinforced concrete

## Abstract

The objective of this work is two-fold. First, we attempt to fit the experimental data on the flexural fatigue of plain and fiber-reinforced concrete with a probabilistic model (Saucedo, Yu, Medeiros, Zhang and Ruiz, Int. J. Fatigue, 2013, 48, 308–318). This model was validated for compressive fatigue at various loading frequencies, but not for flexural fatigue. Since the model is probabilistic, it is not necessarily related to the specific mechanism of fatigue damage, but rather generically explains the fatigue distribution in concrete (plain or reinforced with fibers) for damage under compression, tension or flexion. In this work, more than 100 series of flexural fatigue tests in the literature are fit with excellent results. Since the distribution of monotonic tests was not available in the majority of cases, a two-step procedure is established to estimate the model parameters based solely on fatigue tests. The coefficient of regression was more than 0.90 except for particular cases where not all tests were strictly performed under the same loading conditions, which confirms the applicability of the model to flexural fatigue data analysis. Moreover, the model parameters are closely related to fatigue performance, which demonstrates the predictive capacity of the model. For instance, the scale parameter is related to flexural strength, which improves with the addition of fibers. Similarly, fiber increases the scattering of fatigue life, which is reflected by the decreasing shape parameter.

## 1. Introduction

It is very common for civil infrastructures, the majority of which are made of either plain concrete or reinforced with fibers, to be subjected to time-variable loading [1]. Under such circumstances, the strength of concrete materials under compression [2,3,4,5,6,7,8,9], tension or flexion [10,11,12,13,14,15,16,17,18,19,20,21,22,23] or combined tension-compression [24,25,26] is a great concern. In particular, the addition of fibers combined with high strength concrete (HSC) has improved the tensile or flexural strength of the material, thereby enabling the application of concrete in situations where traditionally, only metallic materials were used. Consequently, the interest in the flexural fatigue strength of concrete has grown significantly, in structures such as bridge slabs, prestressed concrete railroad ties and concrete pavement slabs. However, there is a lack of a systematic analysis of concrete fatigue tests, especially under flexion. In this work, we aim to use the probabilistic model developed by Saucedo et al. [27], originally validated for compressive fatigue, to fit with the experimental data for flexural fatigue in the literature. It should be noted that since the model is probabilistic, rather than mechanical, it is expected to be valid for any type of fatigue loading. Fatigue damage under compression, tension or flexion should be reflected with different model parameters. In other words, the predictive capacity of the model helps to organize, interpret and characterize fatigue tests that would not otherwise be possible. In addition, the model provides a macroscopic vision of fatigue damage behavior, which may lead to further microscopic study of the same phenomenon.

Since the aforementioned model is based on the initial distribution of static material properties, a significant number of static tests (at least ten) is needed in order to extract the initial Weibull distribution. In order to make use of the fatigue data in the literature, in which only three or four tests were usually used to calculate the mean strength, a two-step procedure is developed herein to obtain the model parameters. Subsequently, we briefly summarize the origin and advantages of the fatigue equation used in this work.

For quasi-brittle materials such as concrete, the stress ratio, *R*, the loading frequency, *f*, and the stress level, *S*, play important roles in fatigue behavior [8,28,29,30]. This is in sharp contrast with materials, such as steel, where the effect of the stress range, Δσ, is dominant. Consequently, fatigue models have evolved accordingly to illustrate these effects. Aas-Jakobsen [30] was the first to propose an equation that included the stress-ratio effect as follows:

(1) $$\frac{\sigma_{max}}{\sigma_{c}}=1-\left(1-R\right)\beta \ln N$$

where β is a material parameter, $\sigma_{max}$ the maximum stress, $\sigma_{c}$ the critical stress (material strength) and *N* the number of cycles to failure. This equation was confirmed by Tepfers and Kutti [31] and Tepfers [10] for fatigue strength of concrete in compression and in tension for the splitting tests of cubes. The loading time was first included by Hsu [28] and the loading frequency by Furtak [32] to improve Equation (1). Some authors [33,34] observed the effect of loading frequency on concrete fatigue behavior, but none included it in fatigue equations. In addition, the effect of stress reversal was not included until Zhang et al. [35] redefined the above fatigue equation.

The dispersion in the static properties of concrete was not taken into account until Zhao et al. [36] considered a normal distribution as design codes suggested. Subsequently, a Weibull distribution for static material strength was used by Przybilla et al. [37] for three-point bending tests and by Oh [29] and Li et al. [20] for four-point bending tests, respectively, to fit with the fatigue life of concrete at various stress levels. A probabilistic nine-parameter model was developed by Castillo et al. [38,39] to predict fatigue behavior at any stress level for metallic materials. However, this model does not consider the influence of loading frequency, which has had remarkable effects on the fatigue behavior of concrete specimens. This has led to the development of the probabilistic model of Saucedo et al. [27], which accounts for both the initial distribution of the static material property and the frequency effect of fatigue tests in concrete.

In this work, we first assess the adaptability of the model of Saucedo et al. to the fatigue of plain and steel fiber-reinforced concrete (SFRC) under bending and, thus, obtain highly satisfactory results. For this purpose, a new two-step procedure is developed for parameter estimation; meanwhile, abundant literature data (112 series, which amount to 1350 fatigue tests in total) are collected and analyzed. Those tests cover loading frequencies from 1–20 Hz and the fiber volume ratio until 2.0%. Subsequently, the influence of fiber volume and fiber length on model parameters is discussed. From these results, the capability of the model to reorganize the available fatigue test results is highlighted, thereby permitting an easier interpretation from the model parameters of the flexural fatigue of either plain or fiber-reinforced concrete. Thus, this model becomes one of the few (if not the only) models available in the literature for the prediction of flexural fatigue behavior of plain and fiber-reinforced concrete.

The remainder of this paper is organized as follows. The probabilistic model proposed by Saucedo et al. is summarized in Section 2. The proposed two-step procedure for estimating model parameters is explained in Section 3. The experimental data on flexural fatigue in the literature are analyzed and fitted with the probabilistic model in Section 4. The discussion of the influence of the model parameters is presented in Section 5. Finally, conclusions and recommendations are given in Section 6.

## 2. Probabilistic Model of Saucedo et al.

The probabilistic model of Saucedo et al. is briefly presented below. A more detailed description of this model and its derivation can be found in [27].

The model is reflected by the following expressions:

(2) $$\begin{array}{ccc} PF(\sigma_{f_{0}}) & = & 1-\exp\left[-{\left(\frac{\sigma_{f_{0}}-\sigma_{min_{0}}}{\lambda }\right)}^{k}\right],\;\;\sigma_{f_{0}}\geq \sigma_{min_{0}}, \end{array}$$

(3) $$\begin{array}{ccc} PF(N;\sigma_{max},R,f) & = & 1-\exp\left\{-{\left[\frac{\sigma_{max_{0}}-\sigma_{min_{0}}}{\lambda N^{-a(1-R)}}\right]}^{k}\right\}, \end{array}$$

where $\sigma_{f_{0}}$ is the static strength, either compressive, tensile or flexural (as mentioned, the model was initially assessed for compressive fatigue, but it should also be valid for tensional or flexural since it is probabilistic), with a Weibull distribution, characterized by the scale parameter λ (representative of the static mean strength), the shape parameter *k* (a smaller value indicates a larger scattering) and the location parameter $\sigma_{min_{0}}$. Within the framework of a fatigue distribution, $\sigma_{min0}$ represents the endurance limit, which is the threshold stress below which no fatigue failure will occur. It should be emphasized that the parameters (λ,k) are considered as material properties. Therefore, they are distinct for different types of concrete (plain or reinforced with fibers). In contrast, *a* and α are dependent on both the material type and the loading frequency.

Note that Equation (2) describes the distribution of static properties, whereas Equation (3) represents the probability of failure of a fatigue test under given loading conditions (maximum stress level, loading ratio and loading frequency). In addition, the equivalent static stress level, $\sigma_{max_{0}}$, is related to its dynamic counterpart, $\sigma_{max}$, through the Model Code [40] by means of Equation (4), where $\sigma_{c}^{d}$ and $\sigma_{c}$ are the respective dynamic and static strengths and ${\dot{\sigma }}_{d}$ and ${\dot{\sigma }}_{0}$ are the loading rates of the dynamic and static tests.

(4) $$\frac{\sigma_{c}^{d}}{\sigma_{c}}={\left(\frac{{\dot{\sigma }}_{d}}{{\dot{\sigma }}_{0}}\right)}^{\alpha }$$

When the stress rate, ${\dot{\sigma }}_{d}$, is approximated by:

(5) $${\dot{\sigma }}_{d}=2f\Delta \sigma ,$$

the equivalent static stress level can be related to the maximum stress as follows:

(6) $$\sigma_{max_{0}}=\sigma_{max}{\left(\frac{{\dot{\sigma }}_{0}}{2f\Delta \sigma }\right)}^{\alpha }$$

It needs to be emphasized that even though the exponent α is fitted as a constant equal to 0.014 in the Model Code [40], depending on the available experimental data, it should be adjusted as a function of the loading frequency. Indeed, Saucedo et al. [27] proposed an exponential function to fit with the experimental data of Ruiz et al. [8].

Iso-probability curves for the evolution of the failure stress, $\sigma_{f}$, expressed in Equation (7) were proposed by Saucedo et al. [27] to satisfy the following limit conditions of fatigue strength: (a) the endurance limit is obtained when the fatigue life approaches infinity; (b) the static strength is recovered when the fatigue life is a single cycle or when the stress ratio is equal to one. Operating with Equations (2)–(7), the probabilistic model for compressive fatigue of plain and fiber-reinforced concrete developed by the same authors [27] is acquired as Equation (8).

(7) $$\sigma_{f}=\sigma_{min_{0}}+\left(\sigma_{f_{0}}-\sigma_{min_{0}}\right)N^{-a(1-R)}$$

(8) $$PF\left(N;\sigma_{max},f,R\right)=1-\exp\left\{-{\left[\frac{\sigma_{max}{\left(\frac{{\dot{\sigma }}_{0}}{2f\Delta \sigma }\right)}^{\alpha }-\sigma_{min_{0}}}{\lambda }N^{a(1-R)}\right]}^{k}\right\}$$

Therefore, for fatigue tests carried out under a given loading frequency, the model contains four parameters, (λ,k,a,α), which may be obtained by means of fitting experimental fatigue life data.

## 3. The Two-Step Procedure to Estimate Model Parameters

In order to estimate the model parameters in Equations (2) and (8), the method of maximum-likelihood used by Saucedo et al. [27] was first employed to obtain the parameters λ and *k* for the static material strength distribution and, secondly, to obtain α and *a* for the fatigue life distribution. However, this procedure is only possible if there are sufficient static strength measurements available to obtain the initial distribution. For most of the fatigue test data in the literature, solely the static mean strength and deviation are given. In this scenario, we must carry out the parameter estimation using only the data of the fatigue tests.

Assuming that *n* series of fatigue data are available for different stress levels and/or stress ratios, but with the same loading frequency, the following steps are performed in order to obtain the estimation for (λ,k,a,α). Since the four parameters are for the same material and the same loading frequency, they are enforced to have the same values for all of the series of fatigue tests.

1. For series *i* of fatigue life data, use Mathematica to get the initial estimate ($\lambda_{0}^{i},k_{0}^{i},a_{0}^{i},\alpha_{0}^{i}$), i=1,⋯,n.
2. The set ($\lambda_{0}^{i},k_{0}^{i},a_{0}^{i},\alpha_{0}^{i}$) is input to MATLAB (or any other optimization tool) to obtain the best fit ($\lambda^{i},k^{i},a^{i},\alpha^{i}$).
3. The final estimate for all *n* series is calculated as:
  $$\left(\frac{1}{n}\sum_{i=1}^{n}\lambda^{i},\frac{1}{n}\sum_{i=1}^{n}k^{i},\frac{1}{n}\sum_{i=1}^{n}a^{i},\frac{1}{n}\sum_{i=1}^{n}\alpha^{i}\right)$$

It needs to be highlighted that the endurance limit is preset at 5% of the mean strength to simplify the fitting process and to avoid local maxima.

## 4. Experimental Data on Flexural Fatigue Analyzed through the Probabilistic Fatigue Model

In this section, we explain the experimental data collected on the flexural fatigue of both plain and fiber-reinforced concrete. Brief information on concrete type, fiber content, specimen geometry and fatigue test conditions is summarized herein. Subsequently, the aforementioned procedure is used to fit the fatigue data with the probabilistic model Equation (8). Among the various tests in the literature [10,11,12,13,14,15,16,17,18,19,20,21,22,23,29,35,41,42,43,44], only those with the fatigue life listed in the tables are chosen for our analysis. This is based on the fact that nearly all curves for fatigue life are presented on a logarithmic scale. Consequently, data extracted from those curves will not be as accurate as tabular ones.

The first part deals solely with flexural fatigue tests of plain concrete, whereas the second concerns those of steel fiber-reinforced concrete (SFRC). The test configuration (three- or four-point bending), specimen and fiber dimensions and effective span are collected in Table 1. The measured static properties and the fitted parameters of Equation (8) for each series of experimental data are listed in Table 2 and Table 3, respectively, for plain concrete, whereas those for fiber-reinforced concrete are set forth in Table 4 and Table 5. It needs to be emphasized that, since the static loading rate, ${\dot{\sigma }}_{0}$, is rarely given, a reference value of 0.5 MPa/s is adopted to fit the model parameters in Table 3 and Table 5.

### 4.1. Flexural Fatigue Tests on Plain Concrete Analyzed through the Probabilistic Fatigue Model

In this section, we first give a brief introduction on the flexural fatigue tests of plain concrete carried out by Oh [29], Shi et al. [15] and Zhang et al. [35]. Subsequently, the tabular data are extracted to fit the fatigue Equation (8), the corresponding parameters of which are listed in Table 3. The feasibility of the probabilistic fatigue model is corroborated by the flexural behavior of plain concrete.

#### 4.1.1. Experimental Results of Oh, 1991 [29]

For the purpose of examining the distributions of fatigue life of concrete at various levels of applied stress, Oh [29] performed four-point flexural fatigue tests at three different values of $S_{max}$ (0.65, 0.75 and 0.85), with zero minimum stress. Prismatic concrete beams with dimensions of 500×100×100 mm${}^{3}$ and effective spans of 400 mm, were tested at a rate of 250 cycles/min (4.17 Hz). The static 60-day compressive strength and flexural strength are presented in Table 2. Assuming a two-parameter fatigue life distribution, Oh estimated the mean life as 2240, 27,800 and 410,000 cycles (corresponding to the variations of 28%, 43% and 49%) for the stress levels of 0.85, 0.75 and 0.65, respectively.

**Figure 1:**
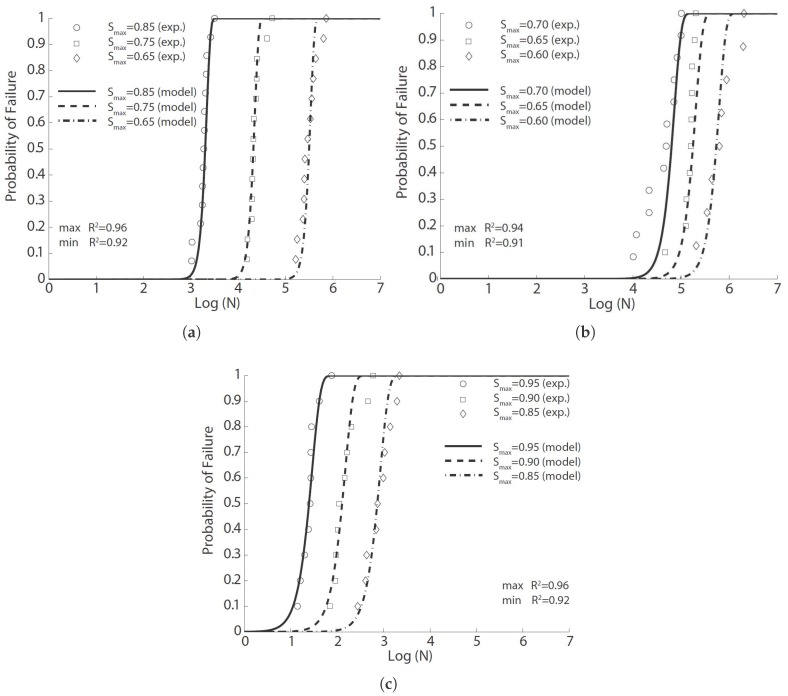
Comparison of the flexural fatigue data for concrete by (**a**) Oh [29], (**b**) Shi et al. [15] and (**c**) Zhang et al. [35], where $R^{2}$ is Pearson’s correlation coefficient.

In this paper, we use the same tabular data given by Oh [29] to fit Equation (8). The result in Figure 1a is obtained with the parameters listed in Table 3. The correlation coefficient varies between 0.92 and 0.96. By setting the probability of failure at 50%, Equation (8) can be inverted to obtain the mean fatigue life, that is 2871, 31,026 and 485,804 cycles for the stress level at 0.85, 0.75 and 0.65, respectively. It is noteworthy that the obtained mean cycles from Equation (8) vary by only 22%, 10% and 18% from those estimated by Oh [29] using three different Weibull distributions for the three levels of fatigue tests. This is strong evidence for the feasibility of Equation (8) to predict the flexural fatigue life of plain concrete at different stress levels.

#### 4.1.2. Experimental Results of Shi et al., 1994 [15]

Shi et al. [15] introduced the concept of equivalent fatigue life, defined as $N^{\left(1-R\right)}$, in the derivation of an expression to describe the flexural fatigue strength of plain concrete. They carried out flexural fatigue tests on 78 plain concrete beams under three-point bending loading. Those beams had a dimension of 500×100×100 mm${}^{3}$, with an effective span of 450 mm. Three different stress ratios (0.08, 0.2 and 0.5) and nine values of stress levels ($S_{max}$ varied from 0.50–0.90, with an increment of 0.05) were examined. We select only those tests with a sufficient number of repetitions, i.e., those with a stress ratio of 0.08, stress levels of 0.60, 0.65 and 0.70 and all at a loading frequency of 1 Hz, to fit Equation (8). The corresponding results are demonstrated in Figure 1b, with the statistical correlation values exceeding 0.91.

It should be emphasized that Oh [29] claimed that the shapes of the probabilistic fatigue-life distributions were dependent on the level of applied stress. From Equation (8), we have observed that this dependency is expressed as the equivalent relation between the static and dynamic stresses. Indeed, the strong correlation of the test data of Shi et al. [15] shown in Figure 1b further verifies this statement. Moreover, the concept of equivalent fatigue life is improved in order to take into consideration the influence of loading frequency on parameter *a* in Equation (8).

#### 4.1.3. Experimental Results of Zhang et al., 1996 [35]

In order to investigate the effects of loading frequency and stress reversal on fatigue properties of plain concrete, Zhang et al. [35] carried out both static and cyclical flexural tests on 171 plain concrete beams under three-point bending configuration. Those beams were of 500×100×100 mm${}^{3}$ in size and 450 mm in effective span. The measured static properties at 28 days and four months are listed in Table 2. Fatigue tests at different stress levels, stress ratios and loading frequencies were carried out. From their own experimental data and those from the literature, Zhang et al. [35] established a fatigue equation to account for the effect of loading frequency and stress reversal. We have chosen only those with a sufficient number of samples to fit with Equation (8). The obtained results are illustrated in Figure 1c for a constant stress ratio (0.2). Note that the correlation coefficient surpasses 0.92 for all three stress levels.

### 4.2. SFRC Flexural Fatigue Tests Analyzed through the Probabilistic Fatigue Model

In this section, we first summarize the flexural fatigue tests carried out by Johnston and Zemp [41], Singh et al. [42], Mohammadi and Kaushik [17] and Goel and Singh [44] for fiber-reinforced concrete. The static strength data are extracted and listed in Table 4. Subsequently, Equation (8) is used to fit with that data. The corresponding parameters are presented in Table 5. The feasibility and predictive nature of the probabilistic fatigue model given by Equation (8) are demonstrated for SFRC.

#### 4.2.1. SFRC Flexural Fatigue Tests by Johnston and Zemp [41]

In the early 1990s, Johnston and Zemp [41] studied the performance of steel fiber-reinforced concrete under flexural fatigue loading in terms of fiber content (0.5%, 1.0% and 1.5% in volume fraction), fiber aspect ratio, $L_{f}/D_{f}$ (47–100) and fiber type. Specifically, eight fiber-reinforced mixtures and one control concrete were cast; five types of straight fibers of uniform cross-section were used: smooth hard drawn wire (SW), slit sheet (SS), hooked-end wire (HW), crimped hard-drawn wire (CW), melt and surface-deformed wire (SDW). In total, 194 fatigue tests and 135 complementary static tests on prismatic beams under the three-point bending configuration were conducted. The beams were 356×102×102 mm${}^{3}$ in size and 305 mm in effective span. Both the first crack strength and the ultimate strength were recorded at the age of 60 days; see Table 4. The fatigue tests were carried out at a loading frequency of 15 Hz up to 0.5 million cycles, and the stress ratio was kept at 0.1. A minimum of six specimens were tested at each stress level from 0.75–0.98. The authors stated that since the static first-crack and ultimate strengths vary by an insignificant amount, the *S*-*N* relationships can be established based on stress level as a percentage of either strength, based on actual applied stress versus the number of loading cycles. Fiber content is the primary governing factor, whereas aspect ratio and fiber type are of secondary importance. The best performance was obtained with 1.5% in the volume ratio and 70 in the aspect ratio of cold drawn wires. The two-million endurance limit varied from 66–82 percent of the static first-crack strength.

**Figure 2:**
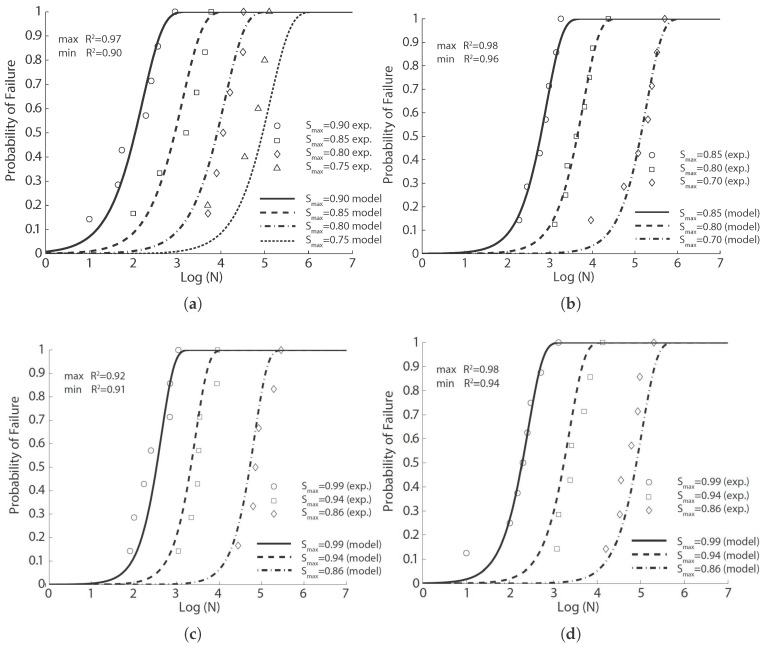
Comparison of flexural fatigue data of Johnston and Zemp [41] with the fitted probabilistic model Equation (8) for concrete reinforced with smooth hard-drawn fibers, aspect ratio of 75: (**a**) 0%; (**b**) 0.5%; (**c**) 1% and (**d**) 1.5% volume ratios.

**Figure 3:**
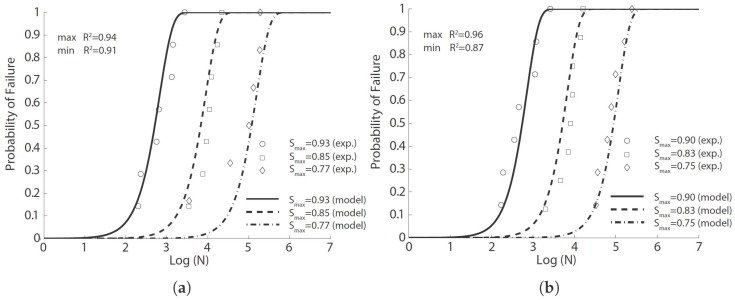

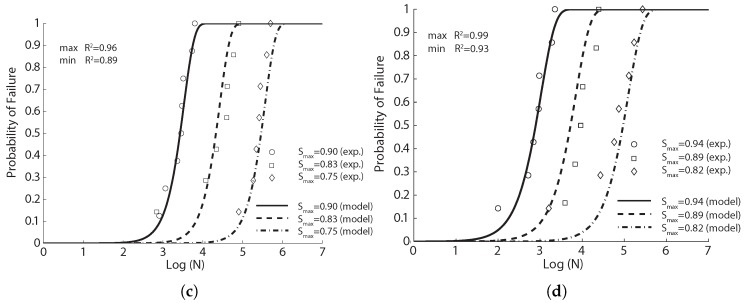
Comparison of flexural fatigue data of Johnston and Zemp [41] with the fitted probabilistic model Equation (8) for concrete reinforced with 1.0% steel fibers of (**a**) SW(50); (**b**) SDW(47); (**c**) ME(54) and (**d**) SS(71) indicated as fiber type(aspect ratio).

We first fit this experimental fatigue data with Equation (8) for the same type of fiber (smooth hard drawn wires, 75 in aspect ratio) and with volume ratios of 0.5%, 1.0% and 1.5%; the corresponding results are illustrated in Figure 2 for each concrete with the model parameters listed in Table 5. Secondly, without distinguishing between the fiber type or the aspect ratio, the fitted results are illustrated in Figure 3 for the same fiber volume ratio (1.0%) using the same set of model parameters listed in Table 5. It is noteworthy that the correlation coefficients surpass 0.90 for the same types of fibers. This coefficient decreases significantly when the fibers are distinct. This indicates that for the same amount of fiber content, even though the flexural strength may not greatly vary, the fatigue life may vary significantly depending on the type of steel fibers.

#### 4.2.2. SFRC Flexural Fatigue Data by Singh et al., 2005 [42]

In order to examine the influences of fiber length and fiber volume fraction, Singh et al. [42] conducted fatigue tests for SFRC reinforced with long and short fibers (50 mm and 25 mm in length), 0.6 mm in width and 0.2 mm in thickness. Nine types of concretes, with three fiber proportions, 65%L-35%S, 50%L-50%S, 35%L-65%S (where L and S represent long and short fibers, respectively), and three fiber volume ratios, 1.0%, 1.5%, 2.0%, were designed. Prismatic specimens with a size of 500×100×100 mm${}^{3}$ were cast and tested. In particular, 269 fatigue tests (20 Hz in loading frequency, stress ratio if zero) and 108 complimentary static ones were carried out under four-point bending configurations. The average compressive and flexural strength of the nine mixes at the age of 28 days are listed in Table 4.

**Figure 4:**
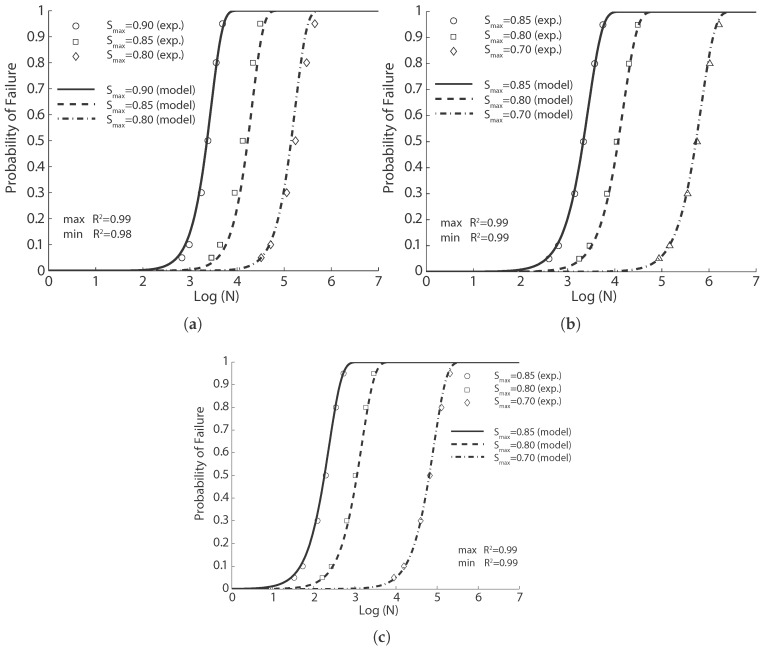
Comparison of the flexural fatigue data of Singh et al. [42] with the fitted probabilistic model Equation (8) for SFRC with volume ratios of (**a**) 1%; (**b**) 1.5% and (**c**) 2%, with 65% of long fibers and 35% of short fibers.

**Figure 5:**
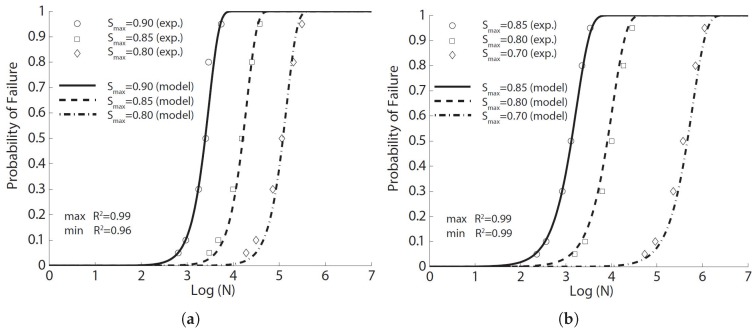

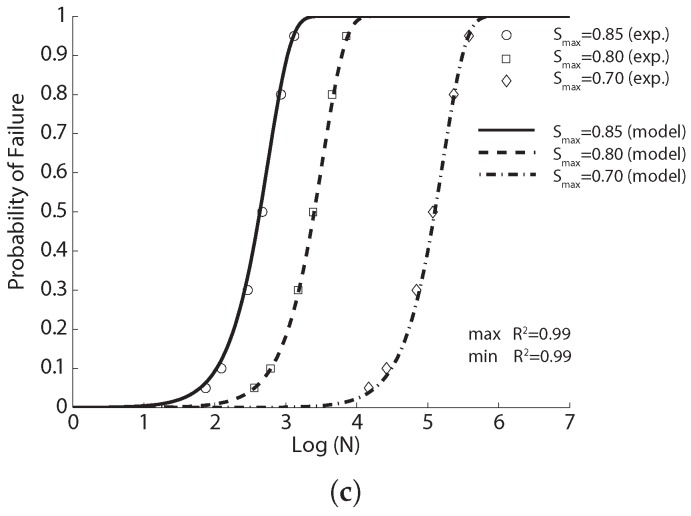
Comparison of the flexural fatigue data of Singh et al. [42] with the fitted probabilistic model Equation (8) for steel fiber-reinforced concrete (SFRC) with volume ratios of (**a**) 1%; (**b**) 1.5% and (**c**) 2%, with equal proportions of long and short fibers.

**Figure 6:**
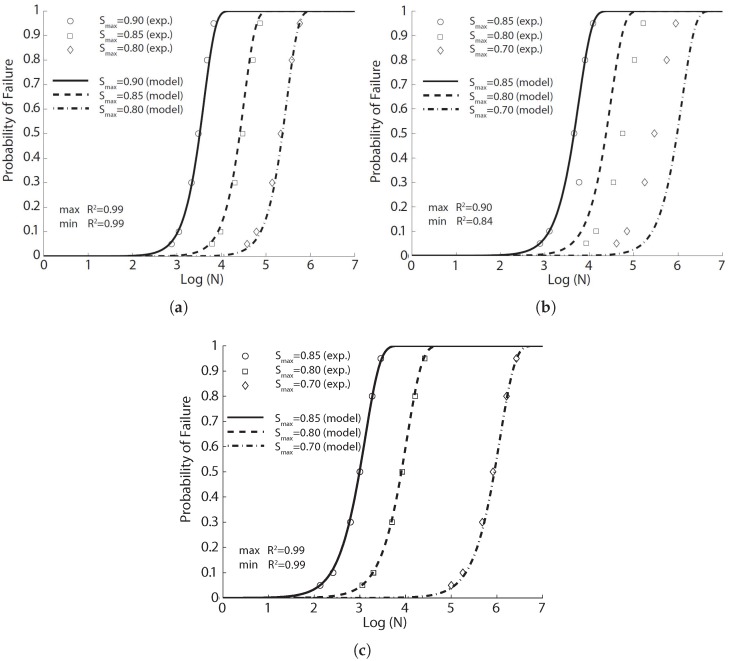
Comparison of the flexural fatigue data of Singh et al. [42] with the fitted probabilistic model Equation (8) for SFRC with volume ratios of (**a**) 1%; (**b**) 1.5% and (**c**) 2%, with 35% of long fibers and 65% of short fibers.

The experimental fatigue data obtained and the fit model curves for each series of tests are shown in Figure 4, Figure 5 and Figure 6 for fiber proportions of 65%L-35%S, 50%L-50%S and 35%L-65%S, respectively. It should be emphasized that Singh et al. [42] concluded that the statistical distribution of equivalent SFRC fatigue-life at a given stress level approximately follows a two-parameter Weibull distribution. From Figure 4, Figure 5 and Figure 6, we observe that the same set of parameters for Equation (8) produces an excellent correlation for three different stress levels. This further indicates the validity of Equation (8) in predicting SFRC fatigue life. This is further demonstrated in Table 6, where the estimated mean fatigue life cycles by Singh et al. [42] by inverting Equation (8) in this paper is shown. In order to have a clear picture of the effect of fiber content, only the common stress level of 80% is selected for this comparison. It is noteworthy that the fatigue life decreases with the increase of the fiber content for the same stress level, $\sigma_{max}/f_{r}$. However, when the maximum stress, $\sigma_{max}$, is maintained, the mean fatigue life is shown to significantly increase with the fiber volume ratio.

#### 4.2.3. SFRC Flexural Fatigue Data by Mohammadi and Kaushik, 2005 [17]

Mohammadi and Kaushik [17] studied the fatigue life of plain and steel fiber-reinforced concrete with different volume fractions (0%, 1%, 1.5%, 2%) and fiber lengths (50 mm and 25 mm) for various levels of the applied fatigue stress. All of the fibers were of the same width (2 mm) and thickness (0.2 mm). Extensive experimental research was planned in which 210 flexural fatigue tests and 84 complementary static ones were conducted on prismatic plain concrete and fibrous concrete specimens of 500×100×100 mm${}^{3}$ in size under three-point loading (450 mm in span). The static properties for seven types of concrete (all sharing the same matrix) at the age of 90 days were measured and listed in Table 4.

**Figure 7:**
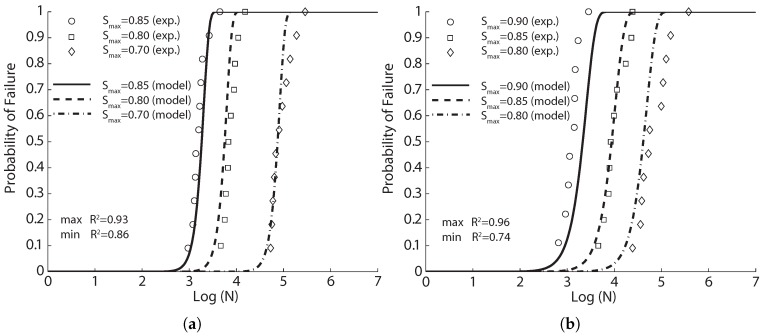

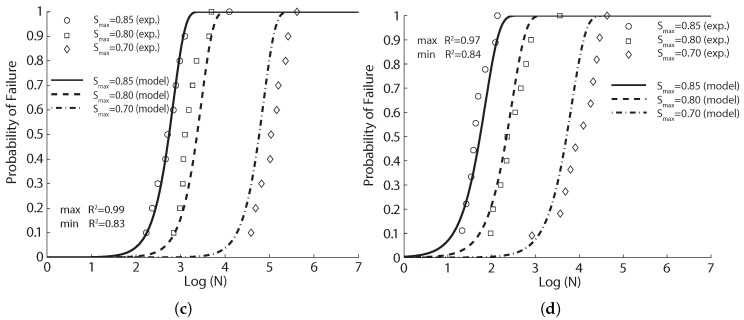
Comparison of the flexural fatigue data of Mohammadi and Kaushik [17] with the fitted probabilistic model Equation (8) for concrete reinforced with fibers of 50 mm in length and volume ratios of (**a**) 0%; (**b**) 1%; (**c**) 1.5% and (**d**) 2%.

**Figure 8:**
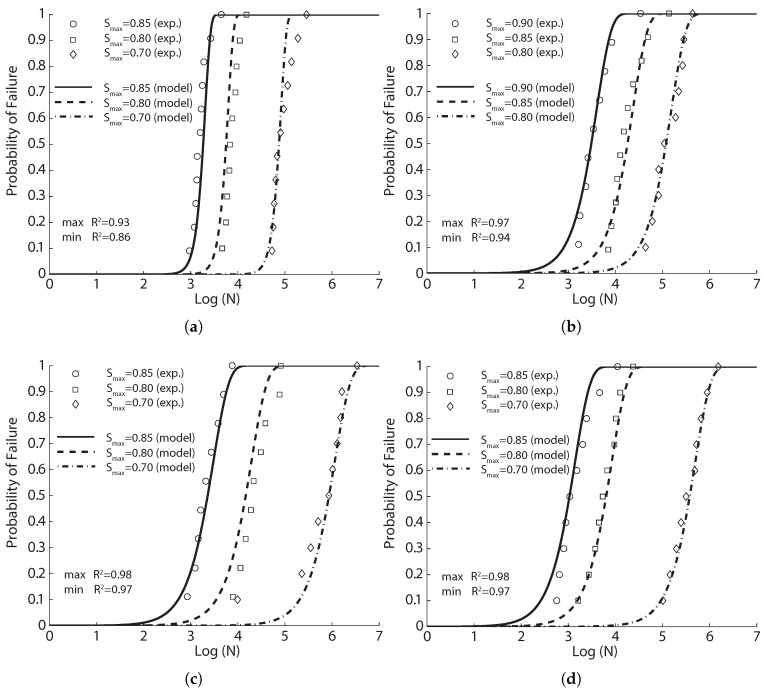
Comparison of the flexural fatigue data of Mohammadi and Kaushik [17] with the fitted probabilistic model Equation (8) for concrete reinforced with fibers of 25 mm in length and volume ratios of (**a**) 0%; (**b**) 1%; (**c**) 1.5% and (**d**) 2%.

The fatigue tests were performed at a loading frequency of 20 Hz. Three stress levels, all with the same stress ratio of 0.1, were used for each concrete. The authors fitted their fatigue data with a two-parameter Weibull model for each stress level with correlation coefficients exceeding 0.90. We adopt Equation (8) herein to get a single fitting for the fatigue tests carried out at three different stress levels, the corresponding results of which are given in Figure 7 and Figure 8 for long (50 mm) and short (25 mm) fibers, respectively. Taking into account the fact that the model parameters for the same material had to be the same, the correlation is excellent.

In Table 7, we compare the mean fatigue life cycles obtained by Mohammadi and Kaushik and by inverting Equation (8) for the stress level of 80%. As expected, the comparison is very strong. The mean fatigue life is observed to decrease as the fiber content increases. Also presented in Table 7 is the predicted mean fatigue life, $N_{f}$, when the maximum stress is set as the flexural strength (5.35 MPa) of the reference plain concrete. Note that the mean fatigue life increases with the fiber content, and this improvement is more pronounced for short fibers.

#### 4.2.4. SFRC Flexural Fatigue Data by Goel and Singh, 2014 [44]

Recently, Goel and Singh carried out a study on the fatigue performance of plain and steel-fiber reinforced self-compacting concrete (SCC). The experimental campaign consisted of 250 flexural fatigue tests and 195 complementary static flexural tests on four-point-bend beams with dimensions of 500×100×100 mm${}^{3}$ and spans of 450 mm. Four types of concrete with the same matrix (the control concrete), the same type of steel fiber (30 mm in length and 1 mm in diameter) and different fiber volume ratios—0%, 0.5%, 1% and 1.5%—were studied. The fatigue tests were performed at a loading frequency of 10 Hz, with the stress ratio kept at 0.1 and the stress level, *S*, varied from 0.65–0.85. The authors used a two-parameter Weibull model to fit the fatigue life distribution for the obtained test data. The shape parameter and characteristic life (or the scale parameter) were determined from the *S*-*N* relationships. The measured static compressive strength, $f_{c}$, the flexural strength, $f_{r}$, at 28 days and the two-million cycle endurance limit, $\sigma_{min_{2m}}$, are listed in Table 4.

**Figure 9:**
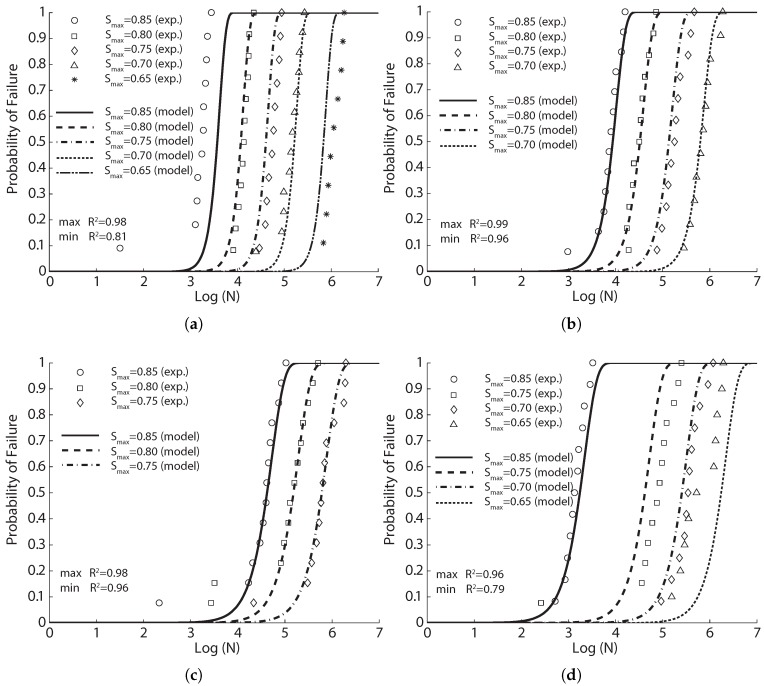
Comparison of the experimental data of Goel and Singh [44] with the fitted probabilistic model Equation (8) for concrete reinforced with steel fibers, (**a**) 0%; (**b**) 0.5%; (**c**) 1.0% and (**d**) 1.5% volume ratios.

The experimental fatigue life data and their corresponding fit with the probabilistic model Equation (8) are provided in Figure 9. Note that Pearson’s correlation coefficient, $R^{2}$, varied from 0.81–0.98 for the plain concrete, from 0.79–0.96 for the concrete with a $V_{f}$ of 1.5%, whereas for $V_{f}$ of 0.5% and 1.0%, stronger correlations were achieved ($R^{2}$ is more than 0.96%). This means that by adding an adequate amount of fibers, on the one hand, there can be a reduction of the dispersion in fatigue life and an improvement in the fatigue performance under flexion. On the other hand, when $V_{f}$ surpasses a certain value, the benefits of fibers may decrease for the same stress level (mainly due to the fact that high fiber content distorts the matrix and produces pores and imperfections in the material that favors the initiation of cracks [45]). This is different from that of static properties as shown in Table 4, where both the compressive and flexural strengths, as well as the two-million endurance limit increased with the fiber volume ratio. This is further demonstrated in Section 4.

In Table 8, we compare the mean fatigue life cycles obtained by Goel and Singh [44] and by inverting Equation (8) for the stress level of 80%. As expected, the comparison is very strong. The mean fatigue life is observed to increase as the fiber content increases until 1.5% for the same stress level. This trend is different from that of Mohammadi and Kaushik [17]. This may be attributed to (a) the different fiber and (b) the lower loading frequency.

Additionally, the predicted mean fatigue life, $N_{f}$, is shown in Table 8, when the maximum stress is set as the flexural strength (4.85 MPa) of the reference plain concrete. Note that the mean fatigue life increases with the fiber content, and this improvement is more pronounced when $V_{f}$ is higher.

## 5. Discussion

As mentioned previously, among the four parameters (k,λ,a,α), a smaller *k* represents a wider scattering; a larger λ corresponds to stronger material strength (in the case of bending, λ is approximately 95% of the flexural resistance, $f_{r}$). The exponent α represents the dynamic amplification of the material strength, whereas *a* reflects the influence of loading frequency, when the others do not change. In other words, both λ and α can be considered material properties, whereas *k* indicates that the static properties are distributive in more or less extension. In this section, we analyze the results obtained in Section 3; specifically, we focus on the influences of the fiber volume ratio $V_{f}$, fiber length, $L_{f}$ and loading frequency, *f*. It should be emphasized that, due to the probabilistic model Equation (8), we are able to analyze and interpret vast experimental data through model parameters in a more organized manner.

### 5.1. The Influence of the Fiber Volume Fraction, $V_{f}$

**Figure 10:**
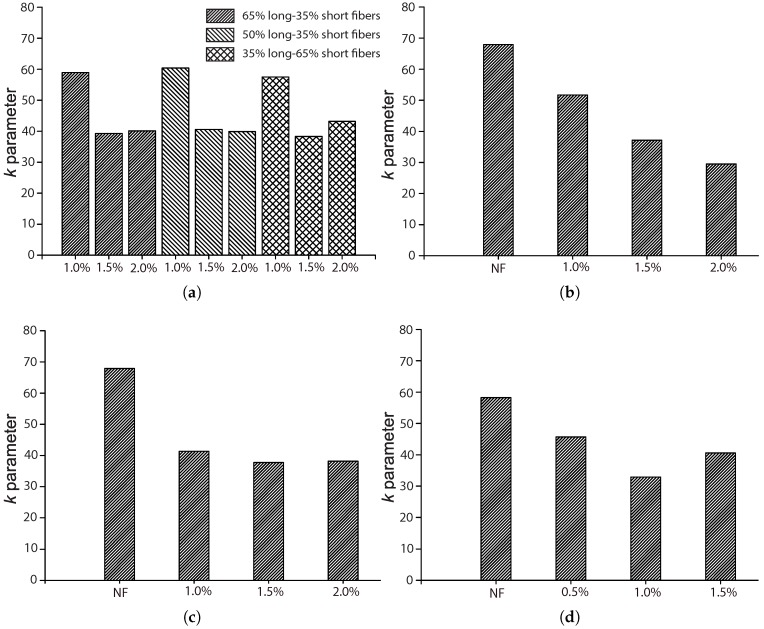
Influence of fiber volume fraction on the shape parameter, *k*: (**a**) Singh et al. [42]; (**b**) Mohammadi and Kaushik [17], 50 mm fiber length; (**c**) Mohammadi and Kaushik [17], 25 mm fiber length; (**d**) Goel and Singh [44].

**Figure 11:**
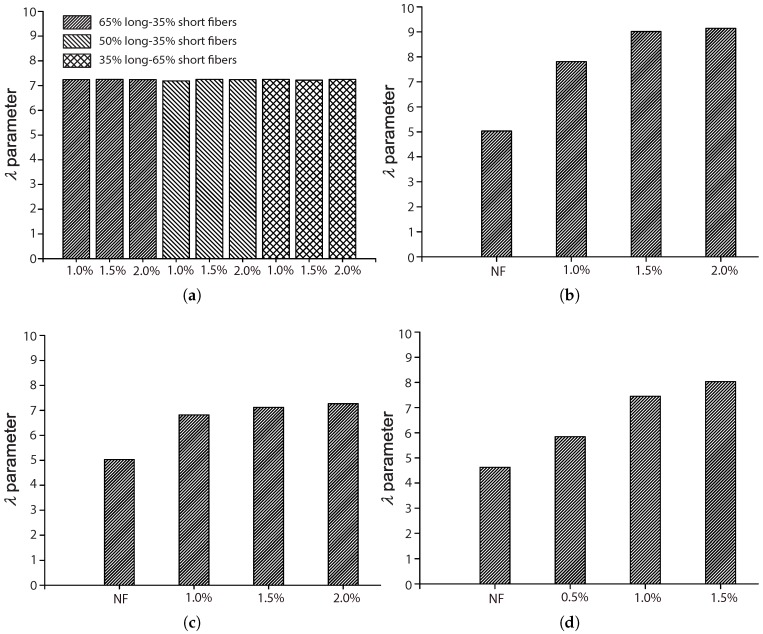
Influence of fiber volume fraction on the scale parameter, λ: (**a**) Singh et al. [42]; (**b**) Mohammadi and Kaushik [17], 50 mm fiber length; (**c**) Mohammadi and Kaushik [17], 25 mm fiber length; (**d**) Goel and Singh [44].

**Figure 12:**
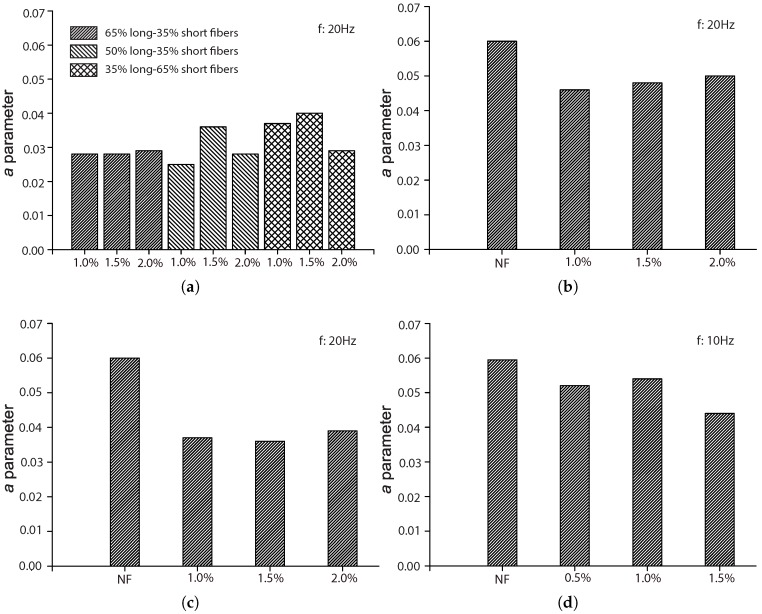
Influence of fiber volume fraction on parameter *a*: (**a**) Singh et al. [42]; (**b**) Mohammadi and Kaushik [17], 50 mm fiber length; (**c**) Mohammadi and Kaushik [17], 25 mm fiber length; (**d**) Goel and Singh [44].

**Figure 13:**
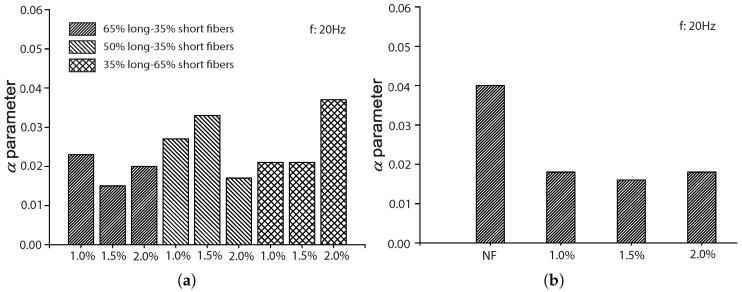

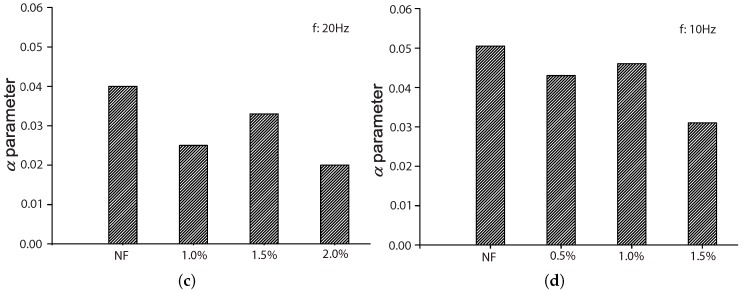
Influence of fiber volume fraction on parameter α: (**a**) Singh et al. [42]; (**b**) Mohammadi and Kaushik [17], 50-mm fibers; (**c**) Mohammadi and Kaushik [17], 25-mm fibers; (**d**) Goel and Singh [44].

Among the four references described in Section 4.2, three of them [17,42,44] explored the influence of the fiber volume ratio by maintaining the same fiber length and concrete matrix. Moreover, except for Mohammadi and Kaushik [17], the complementary tests for plain concrete were also included, which further highlighted the effect of fiber addition. The work of Mohammadi and Kaushik [17] is the only one that considered the mixed proportion of both short and long fibers. Figure 10, Figure 11, Figure 12 and Figure 13 set forth the variations of the parameters (*k*, λ, *a*, α) with respect to the fiber volume ratio, $V_{f}$.

From Figure 10, we observe that *k* is the highest for plain concrete. For the concrete reinforced with fibers of 50 mm in length, 2×0.2 mm${}^{2}$ in the cross-section, *k* decreases with the volume ratio $V_{f}$ for the four values (0.5%, 1.0%, 1.5% and 2.0%) studied by Mohammadi and Kaushik [17]. For the one reinforced with fibers of 25 mm in length and 2×0.2 mm${}^{2}$ in the cross-section, or fibers of 30 mm in length and 1 mm in diameter, tested by Goel and Singh [44], the minimum *k* was reached at 1.0% of $V_{f}$. In the case of mixed proportions of long and short fibers, *k* hardly varies with the mix proportion, while a slight difference is observed for volume ratios of 1.5% and 2.0%. This indicates that fiber addition contributes to the further scattering of fatigue resistance, which is usually attributed to the additional voids created around the fibers [45].

Regarding the scale parameter, λ, we observe from Figure 11 that the minimum is obtained for plain concrete, even though an ascending trend is noticed as $V_{f}$ increases. Only a slight improvement is perceived when $V_{f}$ increased from 1.0% to 1.5% and 2.0%. In the case of hybrid fibers of different lengths, λ does not vary with either the volume fraction or the mix proportion.

From Figure 12, we observe that parameter *a* is the highest for plain concrete. It decreases with the addition of steel fibers, but no significant difference is observed for volume fractions of 0.5%, 1.5% and 2.0%. However, this diminution of *a* was more noticeable when the fatigue tests were carried out at 20 Hz rather than at 10 Hz. This indicates that the effect of fiber addition is stronger when the loading frequency is 20 Hz.

From Figure 13, a trend similar to that of *a* is observed for parameter α. Specifically, the maximum α is reached for plain concrete. The addition of fibers reduces the value of α, but this reduction is not clearly proportional to the amplitude of $V_{f}$, but rather to a higher loading frequency. In other words, the dynamic effect is stronger when the loading frequency is higher, which is as expected.

**Figure 14:**
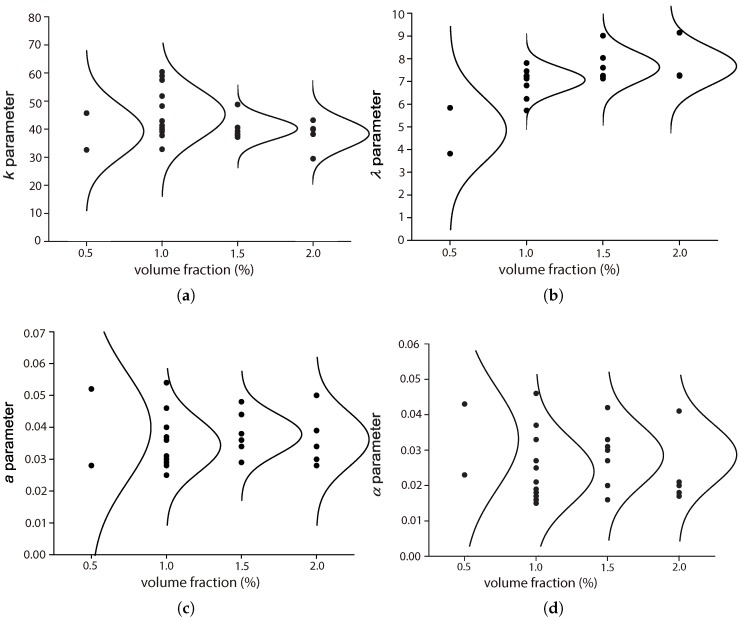
A global perspective of the influence of fiber volume fraction on parameters (**a**) *k*; (**b**) λ; (**c**) *a* and (**d**) α.

In Figure 14, we plot all of the data points shown in Table 3 and Table 5 for the four parameters together with their density distribution. Note that there is a clear ascending trend for λ, which corresponds to the fact that the flexural strength increases with the fiber amount.

### 5.2. The Influence of the Fiber Length, $L_{f}$

**Figure 15:**
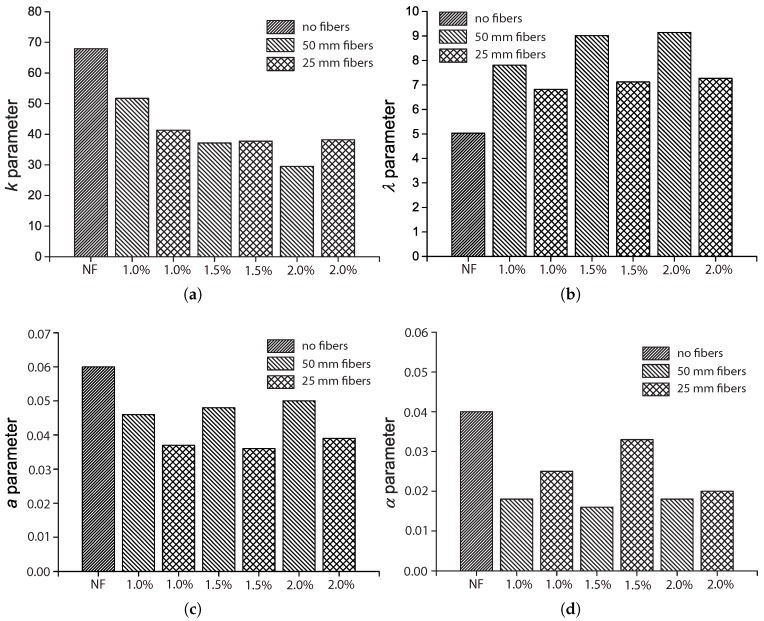
Influence of fiber length on model parameters: (**a**) *k*; (**b**) λ, (**c**) *a* and (**d**) α, by fitting the flexural fatigue tests by Mohammadi and Kaushik [17].

The influence of fiber length $L_{f}$ was only covered by Mohammadi and Kaushik [17] for fibers of 50 mm and 25 mm in length, 2 mm in width and 0.2 mm in thickness. We plot the fitted parameters (k,λ,a,α) by their fatigue tests in Figure 15 with respect to the fiber volume ratio, $V_{f}$.

As mentioned previously, the shape parameter, *k*, is higher for the control concrete due to the fact that the addition of fibers may modify the concrete matrix by creating more voids around the fibers, and consequently, the scattering on the fatigue life increases. This effect results in lower *k* values when the fibers are added. From Figure 15a, it may be observed that the reduction of *k* due to fiber addition has a stronger correlation with $V_{f}$ for long fibers and a weaker correlation for short ones. This can be attributed to the easier workability of short fibers. In other words, a more uniform fiber distribution is achieved during the mixing process when shorter fibers are added.

Figure 15b illustrates the influence of $L_{f}$ on the scale parameter, λ, which is proportional to the flexural strength as far as flexural fatigue tests are concerned. We observe that both long and short fibers improve the material strength by more than 40%. Nevertheless, this improvement is more pronounced for longer fibers. In addition, with a further increase of $V_{f}$ beyond 1.0% (1.5%) for short (long) fibers, strength enhancement may be hardly distinguished.

Figure 15c shows that parameter *a* decreases upon fiber addition. This diminution is more evident for short fibers. Specifically, for 1.0% of the volume fraction of short fibers, *a* is 43% less than that of plain concrete; for 1.0% of long fibers, *a* is 20% less than that of the control concrete.

Regarding α, from Figure 15d, we observe that the highest value is achieved for plain concrete, and a reduction of 50% is obtained for long fibers regardless of volume fraction. Less of a decrease of α is obtained for short fibers. This indicates that the dynamic strength of the SFRC is more sensitive when longer fibers were used; see Equation (4).

### 5.3. The Influence of the Loading Frequency, f

**Figure 16:**
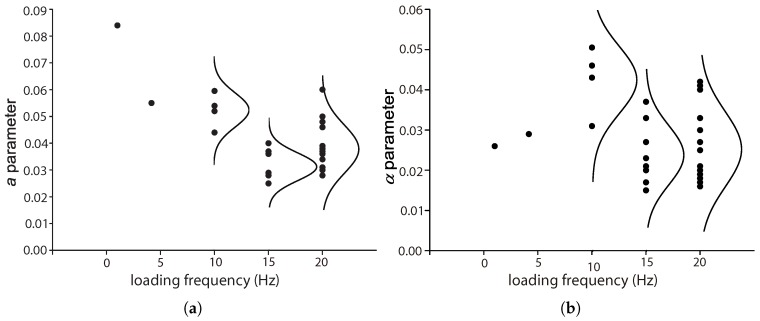
The variations of (**a**) *a* and (**b**) α with respect to the loading frequencies.

The influence of the loading frequency on the compressive fatigue life of plain and reinforced concrete at low loading rates has been demonstrated by the experimental work of Ruiz et al. [8] and Medeiros et al. [9]. This influence is accounted for by the *a* and α parameters in Equation (8) by Saucedo et al. [27]. Figure 16 presents the values for these two parameters for flexural fatigue tests carried out at different loading frequencies for both plain and fiber-reinforced concrete. Note that there is a descending trend for *a* until 15 Hz. For α, a similar trend is observed for loading frequencies between 10 and 15 Hz. Since very few data are available for lower loading frequencies, it is difficult to reach a definitive conclusion.

## 6. Conclusions

We have developed a new two-step procedure in order to estimate the parameters of the model of Saucedo et al. [27] based solely on the fatigue tests and to demonstrate that this probabilistic fatigue model is also valid for predicting fatigue damage under flexion. With only four parameters, the model captures the fatigue damage of plain and fiber-reinforced concrete very well under bending, thereby allowing the analysis of its fatigue behavior through the model parameters. The obtained results conform with well-established conclusions of the fatigue behavior of plain and fiber-reinforced concrete, which further confirms the reliability of the model. Specifically, the following conclusions are drawn.

- With this model, it is feasible to predict the flexural fatigue life of concrete with excellent results. For the four-point flexural fatigue tests carried out by Oh [29], a single set of parameters was fitted with the tests of all three stress levels. The predicted mean cycles are at most 22% different from the ones obtained by Oh. In addition, the reported dependence of fatigue distribution on the stress level is also explained by the probabilistic fatigue model since it fits all of the results with the same model parameters. With respect to SFRC, fitting the results of Johnston and Zemp [41], for the same amount of fiber content, it can be concluded that even though the flexural strength may not vary much, the fatigue life may vary significantly depending on the type of steel fibers, since the fitted model parameters vary significantly.
- From results of the scale parameter λ, the improvement of the SFRC flexural strength by fiber addition is stated, as well as the greater effectiveness of longer fibers. Even though this is physically evident, when reflected by the model parameter, it is further proof of the predictive ability of the probabilistic model.
- The scattering of flexural fatigue data, which increases with the fiber addition, is measured by *k*, the shape parameter. This scattering is more significant for larger volume ratios of longer fibers, but hardly varies with $V_{f}$ for short fibers. Once again, this evidence strengthens the reliability of the model for the prediction of SFRC flexural fatigue.
- As reflected by the model, for the same stress level, $\sigma_{max}/f_{r}$, the mean fatigue life may decrease or increase with the fiber content. However, when the maximum stress, $\sigma_{max}$, is maintained constant, the mean fatigue life increases significantly with the fiber volume ratio. In addition, at the same volume ratio, short fibers offer more enhancement of fatigue life than longer fibers.

## Figures and Tables

**Table 1:** Specimen and fiber dimensions (in mm), test configuration (TPB: three-point bending; FPB: four-point bending), used by Oh [29], Shi et al. [15] and Zhang et al. [35] for plain concrete and Johnston and Zemp [41], Singh et al. [42], Mohammadi and Kaushik [17] and Goel and Singh [44] for those reinforced with fibers.

Ref.	Test	Specimen	Span	$L_{f}$	$L_{f}/D_{f}$
	Configuration	Dimension (mm)	(mm)	(mm)	
[29]	FPB	500×100×100	400	-	-
[15]	TPB	500×100×100	450	-	-
[35]	TPB	500×100×100	450	-	-
[41]	TPB	356×102×102	305	-	47–100
[42]	FPB	500×100×100	450	25, 50	20, 40
[17]	TPB	500×100×100	450	25, 50	20, 40
[44]	TPB	500×100×100	450	30	30

**Table 2:** Measured compressive strength, $f_{c}$, and flexural strength, $f_{r}$, for the plain concrete tested by Oh [29], Shi et al. [15] and Zhang et al. [35].

Ref.	Age	$f_{c}$ (MPa)	$f_{r}$ (MPa)	*E* (GPa)	ν
[29]	60 days	27	4.58	-	-
[15]	28 days	-	5.83	-	-
[35]	28 days	50.7	7.19	-	-
	4 months	57.4	7.88	41.1	0.17

**Table 3:** Fitted model parameters for flexural fatigue tests of plain concrete carried out by Oh [29], Shi et al. [15] and Zhang et al. [35], as well as the testing conditions.

Ref.	*k*	λ (MPa)	*a*	α	*f* (Hz)	*R*
[29]	95.62	4.93	0.055	0.029	4.17	0.00
[15]	37.42	8.75	0.084	0.026	1	0.08
[35]	69.19	7.52	0.0429	0.0167	1	0.20

**Table 4:** Measured flexural strength, $f_{r}$, by Johnston and Zemp [41], Singh et al. [42], Mohammadi and Kaushik [17] and Goel and Singh [44]. The two-million cycle endurance limit $\sigma_{min_{2m}}$ for the plain and fiber-reinforced self-compacting concrete (SCC), Goel and Singh 2014 [44].

$V_{f}$(%)	0.0	0.5	1.0	1.5	2.0	Age (Days)	Ref.
$f_{r}$ (MPa)	4.45	6.19	7.24	8.11	-	60	[41]
$f_{r}$ (MPa)	5.35	-	-	-	-	28	[42]
(65L-35S)	-	-	7.61	9.05	10.05	28	[42]
(50L-50S)	-	-	7.45	8.44	9.15	28	[42]
(35L-65S)	-	-	7.50	7.98	8.44	28	[42]
$f_{r}$ (MPa)	5.35	-	-	-	-	90	[17]
(50 mm-L)	-	-	7.50	9.44	10.72	90	[17]
(25 mm-S)	-	-	7.16	7.73	8.11	90	[17]
$f_{r}$ (MPa)	4.85	6.05	7.20	9.00	-	28	[44]
$\sigma_{min_{2m}}$ (MPa)	3.00	4.30	5.50	6.40	-	28	[44]

**Table 5:** Model parameters and loading frequency, stress ratios for SFRC flexural fatigue tests carried out by Johnston and Zemp 1991 [41], Singh et al. 2005 [42], Mohammadi and Kaushik 2005 [17] and Goel and Singh 2014 [44].

Johnston and Zemp [41]	*k*	λ (MPa)	*a*	α	*f* (Hz)	*R*
$V_{f}=0.0\%$	32.63	3.82	0.028	0.023	15	0.1
$V_{f}=0.5\%$-SW(75)	40.25	6.23	0.028	0.015	15	0.1
$V_{f}=1.0\%$-SW(75)	48.77	7.60	0.029	0.020	15	0.1
$V_{f}=1.5\%$-SW(75)	48.24	7.25	0.025	0.027	15	0.1
$V_{f}=1.0\%$-SW(50)	37.75	7.12	0.036	0.033	15	0.1
$V_{f}=1.0\%$- SW(100)	39.89	7.24	0.028	0.017	15	0.1
$V_{f}=1.0\%$-SDW(47)	39.21	7.22	0.037	0.021	15	0.1
$V_{f}=1.0\%$-ME(54)	40.04	7.22	0.040	0.021	15	0.1
$V_{f}=1.0\%$-SS(71)	42.94	5.72	0.029	0.037	15	0.1
**Singh et al. [42]**	k	λ **(MPa)**	a	α	f **(Hz)**	R
$V_{f}=1.0\%$-(65L-35S)	58.90	7.24	0.030	0.018	20	0.0
$V_{f}=1.5\%$-(65L-35S)	39.27	7.25	0.036	0.042	20	0.0
$V_{f}=2.0\%$-(65L-35S)	41.13	7.24	0.034	0.041	20	0.0
$V_{f}=1.0\%$-(50L-50S)	60.38	7.19	0.031	0.019	20	0.0
$V_{f}=1.5\%$-(50L-50S)	40.54	7.25	0.034	0.027	20	0.0
$V_{f}=2.0\%$-(50L-50S)	39.89	7.24	0.028	0.017	20	0.0
$V_{f}=1.0\%$-(35L-65S)	57.46	7.25	0.028	0.016	20	0.0
$V_{f}=1.5\%$-(35L-65S)	38.33	7.22	0.038	0.030	20	0.0
$V_{f}=2.0\%$-(35L-65S)	43.19	7.25	0.030	0.021	20	0.0
**Moh.and Kaushik [17]**	k	λ **(MPa)**	a	α	f **(Hz)**	R
$V_{f}=0.0\%$	67.90	5.03	0.060	0.040	20	0.1
$V_{f}=1.0\%$-L	51.72	7.81	0.046	0.018	20	0.1
$V_{f}=1.5\%$-L	37.15	9.01	0.048	0.016	20	0.1
$V_{f}=2.0\%$-L	29.48	9.14	0.050	0.018	20	0.1
$V_{f}=1.0\%$-S	41.32	6.82	0.037	0.025	20	0.1
$V_{f}=1.5\%$-S	37.87	6.86	0.038	0.026	20	0.1
$V_{f}=2.0\%$-S	38.18	7.27	0.039	0.020	20	0.1
**Goel and Singh [44]**	k	λ **(MPa)**	a	α	f **(Hz)**	R
$V_{f}=0.0\%$	58.22	4.63	0.060	0.051	10	0.1
$V_{f}=0.5\%$	45.71	5.84	0.052	0.043	10	0.1
$V_{f}=1.0\%$	32.87	7.45	0.054	0.046	10	0.1
$V_{f}=1.5\%$	40.58	8.03	0.044	0.031	10	0.1

**Table 6:** Comparison of the mean fatigue life cycles given by Singh et al. [42] and those predicted by inverting Equation (8) for the tests of Singh et al. [42] at a stress level of 80%. $N_{f}$ is the predicted mean fatigue life where $\sigma_{max}$ is set at the static strength of the plain concrete.

Fiber Volume	$N_{f_{1}}$	$N_{f_{2}}$	$N_{f}$
Ratio-(Mix Proportion)	(Cycles)	(Cycles)	($10^{6}$ Cycles)
$V_{f}=1.0\%$-(65L-35S)	220,736	120,147	11.6
$V_{f}=1.5\%$-(65L-35S)	14,193	14,283	104
$V_{f}=2.0\%$-(65L-35S)	1313	1046	365
$V_{f}=1.0\%$-(50L-50S)	146,313	160,597	6.4
$V_{f}=1.5\%$-(50L-50S)	13,070	8841	12.6
$V_{f}=2.0\%$-(50L-50S)	3174	307	49.2
$V_{f}=1.0\%$-(35L-65S)	276,571	300,055	23.2
$V_{f}=1.5\%$-(35L-65S)	73,675	21,410	2.9
$V_{f}=2.0\%$-(35L-65S)	11,236	7787	30.0

**Table 7:** Comparison of the mean fatigue life cycles given by Mohammadi and Kaushik [17], $N_{f_{1}}$, and those predicted by inverting Equation (8), $N_{f_{2}}$, for the tests in [17] at a stress level of 80%. $N_{f}$ is the predicted mean fatigue life when $\sigma_{max}$ is set at the static strength of the plain concrete.

Concrete	$N_{f_{1}}$	$N_{f_{2}}$	$N_{f}$
Type	(Cycles)	(Cycles)	($10^{6}$ Cycles)
$V_{f}=0.0\%$	9,229	7195	-
$V_{f}=1.0\%$-L	84,177	46,488	0.74
$V_{f}=1.5\%$-L	2284	3609	1.33
$V_{f}=2.0\%$-L	375	230	1.68
$V_{f}=1.0\%$-S	199,315	135,374	1.11
$V_{f}=1.5\%$-S	36,333	17,268	1.38
$V_{f}=2.0\%$-S	9149	6380	1.79

**Table 8:** Comparison of the mean fatigue life cycles given by Goel and Singh, 2014 [44], $N_{f_{1}}$, and those predicted by inverting Equation (8), $N_{f_{2}}$, for the tests in [44] at a stress level of 80%. $N_{f}$ is the predicted mean fatigue life when $\sigma_{max}$ is set at the static strength of the plain concrete (4.85 MPa).

Fiber	$N_{f_{1}}$	$N_{f_{2}}$	$N_{f}$
Volume Ratio	(Cycles)	(Cycles)	($10^{6}$ Cycles)
$V_{f}=0.0\%$	11,808	11,769	-	
$V_{f}=0.5\%$	51,163	30,307	0.03
$V_{f}=1.0\%$	237,492	136,822	5.51
$V_{f}=1.5\%$	83,418	6809	282

## Abbreviations

The following abbreviations are used in this manuscript:

λ,k	scale and shape parameter of a Weibull distribution
α	the exponent that describes the hardening behavior of the material under dynamic loading
β	a material parameter defined by Aas-Jakobsen
$\sigma_{c}$	static material strength
$\sigma_{c}^{d}$	dynamic material strength
${\dot{\sigma }}_{0}$	loading rate employed in a static test
${\dot{\sigma }}_{d}$	loading rate employed in a dynamic test
$\sigma_{f}$	failure stress for a certain fatigue life
$\sigma_{f_{0}}$	static failure stress (material strength)
$\sigma_{max}$	maximum stress
$\sigma_{max_{0}}$	equivalent static stress for $\sigma_{max}$
$\sigma_{min}$	minimum stress
$\sigma_{min_{0}}$	endurance limit
$\sigma_{min_{2m}}$	two-million-cycle endurance limit
Δσ	stress range, $\sigma_{max}$-$\sigma_{min}$
*a*	parameter that adjusts the relation between lnN, *f* and *R*
$D_{f}$	fiber diameter
$f_{c}$	compressive strength
$f_{r}$	flexural strength
$f_{0}$	reference loading frequency
*f*	loading frequency of a fatigue test
$L_{f}$	fiber length
*N*	number of cycles to failure (fatigue life)
$N_{f}$	mean fatigue life (PF = 50%)
PF	probability of failure in any point of the domain $\sigma_{f}$-lnN
*R*	stress ratio defined as $\sigma_{min}$/$\sigma_{max}$
$S_{max}$	applied maximum stress level for a flexural fatigue test, $\sigma_{max}/f_{r}$
$V_{f}$	fiber volume ratio
